# Two colliding epidemics – *obesity* is independently associated with *chronic pain interfering with activities of daily living* in adults 18 years and over; a cross-sectional, population-based study

**DOI:** 10.1186/s12889-016-3696-3

**Published:** 2016-09-30

**Authors:** Sharon A. Allen, Eleonora Dal Grande, Amy P. Abernethy, David C. Currow

**Affiliations:** 1 Southern Adelaide Palliative Services, Repatriation General Hospital, Daw Park, Adelaide, Australia; 2 Population Research and Outcomes Studies Unit, Discipline of Medicine, Health Sciences Faculty, Adelaide University, Adelaide, Australia; 3 Discipline, Palliative and Supportive Services, Flinders University, Bedford Park, Adelaide, Australia; 4 Division of Medical Oncology, Department of Medicine, Duke University Medical Centre, Durham, North Carolina USA

**Keywords:** Pain, Obesity, Period prevalence, Impaired activities of daily living, Population survey

## Abstract

**Background:**

Chronic pain interfering with activities of daily living is highly prevalent in the community. More than 600 million people worldwide are obese. The aim of this paper is to assess if such chronic pain is associated independently with obesity across the adult population, having controlled for other key factors.

**Methods:**

The South Australian Health Omnibus is an annual, population-based, cross-sectional study. Data on 2616 participants were analysed for episodes of daily pain for three of the preceding six months. Obesity was derived from self-reported height and weight. Multivariable logistic regression analysed the associations between chronic pain interfering with activities of daily living, body mass index (BMI) and key socio-demographic factors.

**Results:**

Chronic pain interfering with activities of daily living peaks in people ≥75 years of age while obesity peaks in the 45-54 age group. Pain and obesity together peak in the 55-74 year age group. In the adjusted multinominal logistic regression model, compared to those with no pain, there was a strong association between obesity and *pain that interfered moderately or extremely with day-to-day activities* (OR 2.25; 95 % CI 1.57-3.23; *p* < 0.001) having controlled for respondents’ age, gender, rurality, country of birth and highest educational attainment. People over 65 years of age and those with lower educational levels were more likely to experience such chronic pain related to obesity.

**Conclusion:**

This study demonstrates a strong association between chronic pain and obesity/morbid obesity in the South Australian population. Prospective, longitudinal data are needed to understand the dynamic interaction between these two prevalent conditions.

## Background

Chronic pain is a prevalent problem in today’s society with recent evidence showing the incidence across the community is continuing to rise even in adolescents, with prevalence of between 14-24 % [1]. Incidence increases through the ageing process, with a range reported between 28 and 61 % in elderly adults depending on definitions and the population surveyed [2, 3]. Chronic pain in this current study is defined as ‘an episode of pain most days for more than three of the last six months’ [2].

The World Health Organisation (WHO) report that more than 1.9 billion adults globally were considered overweight with 600 million people identified as obese in 2014. This has more than doubled as a proportion of the world’s population since 1980 [4]. Adding to the concern of the global burden of obesity is that in 2013, 42 million children under the age of 5 were overweight or obese, indicating that the high rate of obesity is set to continue and health-related consequences will be seen in health systems globally. The WHO has also reported that obesity is no longer a problem only in high income countries, with low and middle income countries now facing an obesity epidemic and the health burdens with which obesity is associated [4].

Studies have demonstrated an association between obesity and a variety of chronic pain concerns including musculoskeletal dysfunction, headaches and neuropathic pain [5, 6]. The association of pain and obesity was recently confirmed in a systematic review [7]. Further, where mechanical impacts of obesity may be causing pain, weight loss has been shown to reduce pain subsequently [7]. A recent five year longitudinal study in an elderly cohort of 1099 participants confirmed a relationship between fat mass, body mass index (BMI) and likelihood of experiencing pain [8]. Although fat mass and BMI were both associated with multiple sites of chronic pain, only BMI was associated with lower back pain [8]. This is consistent with an 11 year longitudinal study in the elderly that demonstrated the strong relationship between obesity, chronic disease, ageing and what the authors termed ‘chronic widespread pain’ [9]. Whilst the pathophysiology is unclear, and likely complicated by the multifactorial nature of chronic pain [10], studies have highlighted that patients with both obesity and chronic pain are more likely to experience disability and impact on their activities of daily living, though the mechanisms by which this relationship is mediated are complex and variable [3, 11, 12]. To date, most studies seeking to define the association between chronic pain and obesity do so from the view point of people who have contact with health services (for joint replacement, with chronic pain services or obesity services for example). Many studies are also limited by the age of the cohort studied and, although many studies establish the presence of pain, few outline its impact on day-to-day function by measuring interference with the activities of daily living.

The aim of this study is to examine whether obesity is an independent association of *chronic pain that interferes with activities of daily living* in all adult ages independently of their health service contact, controlling for socioeconomic status, and other demographic factors. The null hypothesis is that there is no association between chronic pain that interferes with activities of daily living and obesity across the population having controlled for key socio-demographic factors.

## Methods

### Setting

South Australia is a state with 7 % of the Australian population and is, on average, slightly older than the rest of the country, and has a slightly lower proportion of people who were born in other countries.

### Sample

Data were collected using the 2006 South Australian Health Omnibus Surveys (HOS) [13, 14]. HOS is a multi-stage, systematic, clustered area sample of households conducted face-to-face annually in spring based on the Australian Bureau of Statistics (ABS) collector districts (CDs). The 2006 HOS sample included households randomly selected from CDs, from the metropolitan Adelaide area and country towns with a population of 1,000 people or more. Within each CD, a random starting point was selected and from this point 10 households were then selected in a given direction with a fixed skip interval. Hotels, motels, hospitals, hostels and other institutions were excluded from the sample. An approach letter introducing the survey was sent to the selected households. One person, aged 15 years and over, who was last to have a birthday, was selected from each household for interview. The interviews were conducted in people’s homes by trained interviewers and up to six call-back visits were made to the chosen households in an attempt to interview the selected person. There was no replacement by another person within the household when the selected member of the household did not want to participate. In total, 2969 people participated, achieving a response rate of 55 %. The data were weighted by five year age group, sex, rurality (metropolitan Adelaide compared to SA country) and household size to the Australian Bureau of Statistics’ 2005 Estimated Residential Population for South Australia to provide population estimates.

### Self-reported pain

Each respondent was asked, over the last six months, if he/she had “an episode of pain that has lasted more than three months”. An episode of pain was defined as “pain experienced on most days” over that period. The following options on a prompt card were given:

1. No
2. Yes, I did get pain but it did not interfere with my day-to-day activities
3. Yes, I did get pain and it interfered a little bit with my day-to-day activities
4. Yes, I did get pain and it interfered moderately with my day-to-day activities
5. Yes, I did get pain and it interfered extremely with my day-to-day activities.

### Data for respondents’ body mass index (BMI)

Body mass index (BMI) was derived from self-reported weight and height with answers accepted in metric or imperial measures. BMI was calculated using the formula, *kilograms/metres*^*2*^, and recoded into three categories (underweight/normal weight, overweight and obese) as defined by WHO [15]. The classification was used for people aged 15 to 17 years and recoded into three categories (normal weight, overweight and obese) [16].

### Socio-demographic measures

Demographic variables included age, sex, rurality (metropolitan/rural), country of birth, highest level of education, marital status, gross annual household income and current work status.

### Statistical analyses

Data analysis was conducted using Statistical Package for Social Sciences (SPSS) for Windows Version 19.0 and Stata Version 13. All estimates and analyses used population weighted data. The analysis was restricted to those who answered questions related to an episode of pain and who provided their height and weight (*n* = 2616). From the five levels of pain categories, respondents were classified into three groups: no pain, pain but no interference (none to a little bit of interference with day-to-day activities) and regular pain that interferes (moderately to extreme interference with day-to-day activities). Univariable analyses compared the proportion of respondents in each of the three pain groups across socio-demographic indicators.

Multinomial logistic regression models were created to analyse ordered episodes of pain (no pain, pain but no interference, or regular pain that interferes) as the dependent variable with three classifications of BMI (normal/underweight; overweight; and obese), adjusting for age group, sex and rurality, highest educational attainment and country of birth. Marital status was notincluded since this variable was highly correlated with other sociodemographic variables (collinearity). Co-morbidities such as osteoporosis, diabetes and arthritis were not included in the regression model as they were considered to be on the causal pathway, and by including these in the model, the association between BMI and episode of pain will largely be attenuated. Possible interaction terms were considered for inclusion in the regression model but were none were considered to be clinically practical.

Ethical approval for the project was obtained from the South Australian Department of Health’s ethics committee. All participants gave verbal informed consent and continued participation in the face-to-face interview was accepted as continued consent.

## Results

**Fig. 1:**
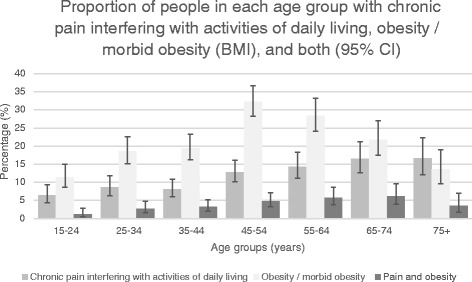
In 2616 randomly selected community members, proportions with chronic pain interfering with activities of daily living, obesity and both

One quarter of the respondents (24.6 %; *n* = 664) experienced an episode of pain that lasted more than three of the last six months (Table 1). Of those people, 54.3 % had pain that did not interfere or only interfered a little bit with their daily activities and 45.7 % had pain that interfered moderately (25.6 % or 6.5 % of the total population) or extremely (*n* = 18.7 % or 4.7 % of the total population) with their daily activities. Chronic pain interfering with activities of daily living peaked in the population over 75 years of age (Fig. 1).

**Table 1:** Prevalence and sample sizes for episode of pain, BMI and covariates

	Analysis sample (*n* = 2616)	
Episode of pain that has lasted more than 3 months		
No	1972	75.4 (73.7–77.0)
Yes, not interfere with day-to-day activities	162	6.2 (5.3–7.2)
Yes, interfere a little bit with day-to-day activities	188	7.2 (6.3–8.3)
Yes, interfere moderately with day-to-day activities	170	6.5 (5.6–7.5)
Yes, interfere extremely with day-to-day activities	124	4.7 (4.0–5.6)
Body mass index		
Normal/underweight	1204	46.0 (44.1–48.0)
Overweight	848	32.4 (30.7–34.2)
Obese	564	21.6 (20.0–23.2)
Demographics characteristics		
Sex		
Male	1326	50.7 (48.8–52.6)
Female	1290	49.3 (47.4–51.2)
Age		
15-24	385	14.7 (13.4–16.1)
25-34	409	15.6 (14.3–17.1)
35-44	483	18.5 (17.0–20.0)
45-54	477	18.2 (16.8–19.8)
55-64	372	14.2 (12.9–15.6)
65-74	286	10.9 (9.8–12.2)
75+	203	7.7 (6.8–8.8)
Area of residence		
Metropolitan	1952	74.9 (73.2–76.5)
Regional	656	25.1 (23.5–26.9)
Marital status		
Married/De Facto	1701	65.0 (63.2–66.8)
Separated/Divorced	211	8.1 (7.1–9.2)
Widowed	134	5.1 (4.3–6.0)
Never Married	561	21.4 (19.9–23.0)
Not stated	10	0.4 (0.2–0.7)
Country of birth		
Australia	1906	72.8 (71.1–74.5)
UK/Ireland	327	12.5 (11.3–13.8)
Other	384	14.7 (13.4–16.1)
Educational attainment		
Up to secondary	1145	43.8 (41.9–45.7)
Trade, Apprenticeship, Certificate, Diploma	995	38.0 (36.2–39.9)
Degree or higher	467	17.9 (16.4–19.4)
Not stated	9	0.4 (0.2–0.7)
Employment status		
Work full or part time	1516	57.9 (56.0–59.8)
Home Duties	231	8.8 (7.8–10.0)
Unemployed	55	2.1 (1.6–2.7)
Retired	516	19.7 (18.2–21.3)
Student	175	6.7 (5.8–7.7)
Not working because work related injury or disability, other	124	4.7 (4.0–5.6)
Annual household income		
Up to $20,000	330	12.6 (11.4–13.9)
$20,001 to $50,000	625	23.9 (22.3–25.6)
$50,001 to $80,000	539	20.6 (19.1–22.2)
$80,001 or more	644	24.6 (23.0–26.3)
Not stated	478	18.3 (16.8–19.8)

2616 participants from the 2006 South Australian Omnibus Survey met the inclusion criteria for this study. 24.6 % of respondents (*n* = 664) reported an episode of pain with a duration of more than three months within the 6 months prior to time of survey. Of the respondents who reported pain, 54.3 % (*n* = 350) stated their pain either did not interfere or only slightly interfered with their activities of daily living. 45.7 % of respondents reporting pain (*n* = 294) stated their pain either moderately or extremely interfered with their activities of daily living

Three pain groups were compared by socio-demographic indicators and health conditions (Table 2). Generally, respondents experiencing *pain but no interference with their daily lives* were more likely to be female, in the older age groups, widowed, and diagnosed with co-morbid illnesses. Respondents experiencing regular *pain that interfered with their daily lives* were more likely to be: obese; older; separated, divorced or widowed; having lower educational qualifications; living in a household with lower incomes; and diagnosed with current comorbid conditions.

**Table 2:** Univariable analyses of severity of pain by BMI and covariates (socio-demographic and health-related variables)

	Total	No pain		Pain but no interference with day-to-day activities		Regular pain that interferes with day-to-day activities	
	*n*	*n*	% (95 % CI)	n	% (95 % CI)	n	% (95 % CI)
Overall	2616	1972	75.4 (73.7–77.0)	350	13.4 (12.1–14.7)	294	11.2 (10.1–12.5)
BMI							
Normal/underweight	1209	961	79.5 (77.1–81.6)	151	12.5 (10.8–14.5)	97	8.0 (6.6–9.7)
Overweight	843	632	74.9 (71.9–77.7)	114	13.6 (11.4–16.0)	97	11.5 (9.5–13.8)
Obese	564	379	67.3 (63.3–71.0)	84	14.9 (12.2–18.1)	100	17.8 (14.9–21.2)
*Covariates*							
Sex							
Male	1326	1026	77.4 (75.0–79.5)	155	11.7 (10.1–13.5)	145	10.9 (9.4–12.7)
Female	290	946	73.3 (70.8–75.7)	195	15.1 (13.3–17.2)	149	11.6 (9.9–13.4)
Age group							
15-24	385	331	85.9 (82.1–89.1)	30	7.7 (5.4–10.8)	25	6.4 (4.3–9.3)
25-34	409	315	76.9 (72.6–80.7)	59	14.5 (11.4–18.2)	35	8.7 (6.3–11.8)
35-44	483	391	80.8 (77.0–84.1)	54	11.1 (8.6–14.3)	39	8.1 (6.0–10.8)
45-54	477	352	73.8 (69.6–77.5)	64	13.4 (10.6–16.8)	61	12.8 (10.1–16.1)
55-64	372	259	69.6 (64.7–74.0)	60	16.1 (12.7–20.2)	53	14.3 (11.2–18.3)
65-74	286	188	65.6 (59.9–70.9)	51	17.9 (13.9–22.8)	47	16.4 (12.6–21.2)
75+	203	137	67.5 (60.8–73.6)	32	15.8 (11.5–21.5)	34	16.6 (12.1–22.3)
Area of residence (rurality)							
Metro. Adelaide	1952	1471	75.4 (73.4–77.2)	273	14.0 (12.5–15.6)	208	10.7 (9.4–12.1)
SA Country	656	496	75.6 (72.1–78.7)	75	11.5 (9.2–14.1)	85	13.0 (10.6–15.8)
Marital status							
Married/De facto	1701	1278	75.2 (73.1–77.2)	234	13.7 (12.2–15.4)	189	11.1 (9.7–12.7)
Separated/Divorced	211	147	69.8 (63.3–75.6)	28	13.4 (9.4–18.6)	35	16.8 (12.4–22.5)
Widowed	34	90	67.1 (58.7–74.4)	22	16.5 (11.1–23.7)	22	16.5 (11.1–23.7)
Never married	561	447	79.7 (76.2–82.8)	66	11.8 (9.4–14.8)	48	8.5 (6.5–11.1)
Country of birth							
Australia	1906	1456	76.4 (74.4–78.2)	252	13.2 (11.8–14.8)	198	10.4 (9.1–11.9)
UK and Ireland	327	228	69.7 (64.5–74.4)	49	15.1 (11.6–19.4)	50	15.2 (11.7–19.5)
Other	384	289	75.3 (70.7–79.3)	49	12.7 (9.8–16.4)	46	12.0 (9.1–15.7)
Educational attainment							
Up to secondary	145	862	75.3 (72.7–77.7)	148	12.9 (11.1–15.0)	135	11.8 (10.1–13.8)
Trade, Apprenticeship, Certificate, Diploma	995	725	72.8 (70.0–75.5)	145	14.6 (12.6–16.9)	125	12.5 (10.6–14.8)
Degree or higher	467	384	82.1 (78.4–85.3)	56	12.0 (9.4–15.3)	28	5.9 (4.1–8.4)
Household annual income							
Up to $20,000	330	216	65.4 (60.1–70.3)	52	15.9 (12.4–20.2)	62	18.7 (14.9–23.3)
$20,001 to $50,000	625	451	72.1 (68.4–75.4)	87	14.0 (11.5–16.9)	87	14.0 (11.5–16.9)
$50,001 to $80,000	539	430	79.7 (76.1–82.9)	66	12.2 (9.7–15.3)	43	8.0 (6.0–10.6)
$80,001 or more	644	495	76.8 (73.4–79.9)	96	15.0 (12.4–17.9)	53	8.2 (6.4–10.6)
Not stated	478	381	79.8 (75.9–83.1)	48	10.0 (7.6–13.0)	49	10.2 (7.8–13.3)
Overall	2616	1972	75.4 (73.7–77.0)	350	13.4 (12.1–14.7)	294	11.2 (10.1–12.5)

Respondents were placed into one of three groups indicating if they experienced pain and if the pain impacted on activities of daily living. Respondents reporting pain without an impact on their activities of daily living were more likely to be female in an older age group, widowed and experiencing co-morbid illness. Respondents who reported regular pain interfering with their activities of daily living were more likely to be from an older age group, obese, living in a lower income household and with a lower educational qualification. This group of respondents were also more likely to be living with active comorbid illnesses

Obesity and morbid obesity was seen in 21.6 % (*n* = 564) of the population. By age group, obesity peaked in the 45-54 year old age group where one third of all respondents were obese or morbidly obese.

In the adjusted multinominal logistic regression model (Table 3), compared to those with no pain, there was a strong association between obesity and *pain that interfered moderately or extremely with day-to-day activities* (OR 2.25; 95 % CI 1.57, 3.23; *p* < 0.001)

**Table 3:** Multinomial logistic regression of severity of pain by BMI

	Unadjusted		Adjusted*	
	OR (95 % CI)	*P* value	OR (95 % CI)	*P* value
No pain (reference)				
Have pain, no interference to a little bit with day-to-day activities				
BMI				
Normal/underweight	1.00		1.00	
Overweight	1.13 (0.85–1.52)	0.395	1.04 (0.77–1.40)	0.803
Obese	1.40 (1.02–1.94)	0.040	1.30 (0.94–1.81)	0.115
Sex				
Male	1.00		1.00	
Female	1.34 (1.05–1.71)	0.020	1.38 (1.06–1.79)	0.016
Age group				
18 to 24	1.00		1.00	
25 to 34	1.68 (1.03–2.75)	0.039	1.68 (0.96–2.95)	0.070
45 to 64	2.20 (1.36–3.54)	0.001	2.10 (1.23–3.61)	0.007
65+	2.79 (1.72–4.54)	<0.001	2.71 (1.58–4.64)	<0.001
Area of residence (rurality)				
SA Country	1.00		1.00	
Metropolitan Adelaide	1.17 (0.87–1.59)	0.296	1.33 (0.94–1.87)	0.105
Educational attainment				
Degree or higher	1.00		1.00	
Up to secondary	1.18 (0.84–1.68)	0.341	1.12 (0.76–1.65)	0.565
Trade, Apprenticeship, Certificate, Diploma	1.38 (0.96–1.97)	0.079	1.35 (0.92–1.96)	0.120
Country of birth				
Australia	1.00		1.00	
UK/Ireland	1.33 (0.94–1.86)	0.105	1.03 (0.72–1.47)	0.890
Other	1.11 (0.78–1.58)	0.574	0.85 (0.59–1.24)	0.402
Have pain, interfere moderately to extremely with day-to-day activities				
BMI				
Normal/underweight	1.00		1.00	
Overweight	1.50 (1.07–2.11)	0.018	1.34 (0.93–1.92)	0.114
Obese	2.61 (1.84–3.71)	<0.001	2.25 (1.57–3.23)	<0.001
Sex				
Male	1.00		1.00	
Female	1.12 (0.86–1.46)	0.392	1.11 (0.83–1.48)	0.475
Age group				
18 to 24	1.00		1.00	
25 to 34	1.62 (0.95–2.76)	0.075	1.30 (0.72–2.35)	0.382
45 to 64	2.70 (1.62–4.51)	<0.001	1.94 (1.10–3.41)	0.022
65+	3.59 (2.10–6.14)	<0.001	2.69 (1.45–4.97)	0.002
Area of residence (rurality)				
SA Country	1.00		1.00	
Metropolitan Adelaide	0.81 (0.59–1.11)	0.194	0.95 (0.66–1.36)	0.771
Educational attainment				
Degree or higher	1.00		1.00	
Up to secondary	1.90 (1.23–2.95)	0.004	1.87 (1.15–3.01)	0.011
Trade, Apprenticeship, Certificate, Diploma	2.39 (1.52–3.75)	<0.001	2.17 (1.35–3.51)	0.001
Country of birth				
Australia	1.00		1.00	
UK/Ireland	1.53 (1.06–2.21)	0.022	1.30 (0.89–1.92)	0.178
Other	1.27 (0.90–1.80)	0.181	1.16 (0.80–1.68)	0.445

The adjusted multinominal logistic regression model demonstrates a strong association between obesity and pain interfering either moderately or extremely with activities of daily living (OR 2.25; 95 % CI 1.57-3.23; *p* < 0.001). When reviewing the association between obesity and pain with minimal to no interference of activities of daily living no association was identified

*BMI* Body mass index, *OR* odds ratio, *CI* confidence interval

*Adjusted by sex, age, area of residence and educational attainment

## Discussion

This study demonstrated that people who were obese were more than twice as likely to have pain that interfered moderately or extremely with activities of daily living having controlled for key socio-demographic factors. Importantly, the rate of pain interfering with activities of daily living is consistent with a previous study of pain interfering extremely with activities of daily living with a quoted rate from the same state of Australia of 5.0 % [17]. A recently published cross-sectional population study of 2508 people from Germany demonstrated very similar rates of chronic pain interfering with activities of daily living (7.3 %; 95 % CI 5.9 %, 8.7 %) and the odds ratio (2.14; 95 % CI 5.9 %, 7.8 %) for an association with obesity [18].

When reviewing the association between BMI and chronic pain, we distinguished between being overweight and being obese and, likewise, between pain that did and did not interfere with people’s activities of daily living. For less troublesome pain, there was no association with being overweight nor obese (Table 3). These findings are consistent with other studies key studies [19–21].

Most population studies in this field highlight trends that chronic pain is more likely to be reported in females, elderly participants and those in lower socio-economic settings, reflecting these findings from South Australia. In a recent nine country, cross-sectional study, all 9 countries demonstrated higher education and wealth as a protective factor in chronic pain in the setting of obesity [22]. Interestingly, the female and elderly respondents in our study were more likely to report pain without significant impact on activities of daily living which differs from the findings of the Olmsted study that demonstrated trends in both females and elderly respondents being more likely to report pain impacting on their daily activities of living [3].

Direct correlation and comparison to previous studies is difficult due to the lack of standardisation of the definitional issues with chronic pain which varies from 4 to 12 weeks and researchers’ approaches to defining pain severity groupings with a minority reporting the impact on activities of daily living. Furthermore there is considerable difference in population age groups which may affect the overall percentage of individuals reporting significant pain.

Population-based studies can only establish association, not causality. In the dyad of pain that interferes with activities of daily living and obesity, either could initiate the cycle of one worsening the other progressively. In studies of people with both, it is apparent that the model of pain limiting mobility thereby worsening weight control in turn leading to worsening pain or obesity generating pain, limiting mobility and thereby worsening obesity is potentially far too simplistic. A study of people with pain suggests that stress-induced eating is a consequence, in part, of catastrophizing about the longer term outcomes of chronic pain [23]. This may mean that any model seeking to define causes-and-effects is likely to be a complex relationship that requires careful consideration of all the contributing factors. In the setting of women with frequent migraines and obesity, catastrophizing, more frequent and more intense migraines were much more likely than in women who did not have obesity [24].

### Limitations of the study

Our data were collected through self-reported surveys which have several limitations. Firstly, this form of data collection is potentially subject to recall bias, however the bias is consistently measured across the population and the duration of recall sought (6 months) is relatively short. Secondly the calculation of BMI for the study was through self-reported weight and height. Studies indicate that individuals may under-report their weight and over-estimate their height leading to likely systematic underestimation of overweight and obesity in this study [13]. Further to this, we have not identified characteristics of adiposity [21, 25, 26].

Our survey could not explore any links between chronic pain and other associated socio-demographic factors including depression, type of employment, smoking/alcohol status, cultural/ethnic background, private health insurance status or history of abuse/violence [2, 11, 12, 22, 26–28].

During this study, we did not approach individuals in hospitals or residential care facilities however we recognise that there is likely to be a large burden of chronic pain in these particular populations.

### Further research

Our study provides supporting evidence to the growing body of research on the prevalence of chronic pain in the Australian population associated with obesity. Recent articles suggesting multifactorial pathogenesis of chronic pain in the obese population (specifically inflammatory mechanisms) would be better supported with further research isolating specific locations of pain (non-weight bearing joints compared with weight bearing joints, upper compared to lower extremity pain) which we have not reviewed as part of our current study [10, 19, 20].

Furthermore, more detailed assessment of attributes of obesity i.e. waist circumference and calculation of fat mass/free fat mass may help to further the understanding of the aetiology of this combination of symptoms – does one usually precede the other or not? [21, 25] More research into the confounding and contributing co-morbidities would be worth further exploration, specifically underlying mental health issues including depression which has been highlighted in multiple studies to have a contributing effect on chronic pain [28].

Finally, longitudinal studies would be beneficial to further our understanding of the genesis and resolution of chronic pain in our society and the impact on activities of daily living over time. For how many people is the obesity a consequence of chronic pain and vice versa?

Clinically, given evidence that weight loss can improve the management of osteoarthritis and its pain (with an aim of delaying or avoiding joint replacement therapy), understanding the genesis of the combination of pain and obesity, and recognising its presence early is a crucial role for all medical practitioners [29].

## Conclusion

There is a strong association between chronic pain and obesity in the South Australian population with individuals identified as obese reporting pain interfering with daily activities twice as frequently as the rest of the population. Direct comparison of the findings of the South Australian population to similar studies is difficult due to lack of standardisation of populations and definitions in other key studies on this topic. The South Australian cohort did have similar trends of socio-economic advantage and higher educational status being protective factors while, at a population level, being identified as female, elderly and with a lower socio-economic demographic increased the likelihood of reporting chronic pain with obesity. Prospective, longitudinal data are needed to understand the dynamic interactions between these two prevalent risk health states in order to understand better their interplay.

## Abbreviations

ABS: Australian Bureau of Statistics
BMI: Body mass index
CD: Collector Districts
CI: Confidence Interval
HOS: Health Omnibus Survey
OR: Odds Ratio
SPSS: Statistical Package for Social Sciences
WHO: World Health Organisation
